# Current and future requirements to industrial analytical infrastructure—part 2: smart sensors

**DOI:** 10.1007/s00216-020-02421-1

**Published:** 2020-02-14

**Authors:** Tobias Eifert, Kristina Eisen, Michael Maiwald, Christoph Herwig

**Affiliations:** 1 Arbeitskreis Prozessanalytik, Gesellschaft Deutscher Chemiker, 60486 Frankfurt am Main, Germany; 2 Covestro Deutschland AG, /Uerdingen, 47829 Krefeld, Germany; 3 Daiichi Sankyo Europe GmbH, 81379 Munich, Germany; 4 Bundesanstalt für Materialforschung und -prüfung (BAM), 12489 Berlin, Germany; 5 ICEBE, Research Area Biochemical Engineering, TU Wien, 1060 Vienna, Austria

**Keywords:** Smart sensors, Industry 4.0, Digital twins, Process intelligence, Process analytical technology, Physical twin, Cyber-physical system

## Abstract

Complex processes meet and need Industry 4.0 capabilities. Shorter product cycles, flexible production needs, and direct assessment of product quality attributes and raw material attributes call for an increased need of new process analytical technologies (PAT) concepts. While individual PAT tools may be available since decades, we need holistic concepts to fulfill above industrial needs. In this series of two contributions, we want to present a combined view on the future of PAT (process analytical technology), which is projected in smart labs (Part 1) and smart sensors (Part 2). Part 2 of this feature article series describes the future functionality as well as the ingredients of a smart sensor aiming to eventually fuel full PAT functionality. The smart sensor consists of (i) chemical and process information in the physical twin by smart field devices, by measuring multiple components, and is fully connected in the IIoT 4.0 environment. In addition, (ii) it includes process intelligence in the digital twin, as to being able to generate knowledge from multi-sensor and multi-dimensional data. The cyber-physical system (CPS) combines both elements mentioned above and allows the smart sensor to be self-calibrating and self-optimizing. It maintains its operation autonomously. Furthermore, it allows—as central PAT enabler—a flexible but also target-oriented predictive control strategy and efficient process development and can compensate variations of the process and raw material attributes. Future cyber-physical production systems—like smart sensors—consist of the fusion of two main pillars, the physical and the digital twins. We discuss the individual elements of both pillars, such as connectivity, and chemical analytics on the one hand as well as hybrid models and knowledge workflows on the other. Finally, we discuss its integration needs in a CPS in order to allow its versatile deployment in efficient process development and advanced optimum predictive process control.

## Introduction

The ability of the process industry and its suppliers to sustainably deliver high-quality products at competitive prices and quickly adapt to evolving customer needs will be crucial to the future competitiveness of the process industry and its suppliers. For this reason, competitiveness means safeguarding the required product quality while making ideal use of equipment, raw materials, and energy.

Process analytical technology (PAT) is particularly valuable for chemical production and manufacturing, especially in the pharmaceutical, food, and (petro)chemical industries. Such tools can be used in the production process for controlling a process as well as for quality assurance of an end product in order to meet the required product specifications as they provide dynamic information about product properties, material flow properties, or operating conditions [1, 2].

**Fig. 1:**
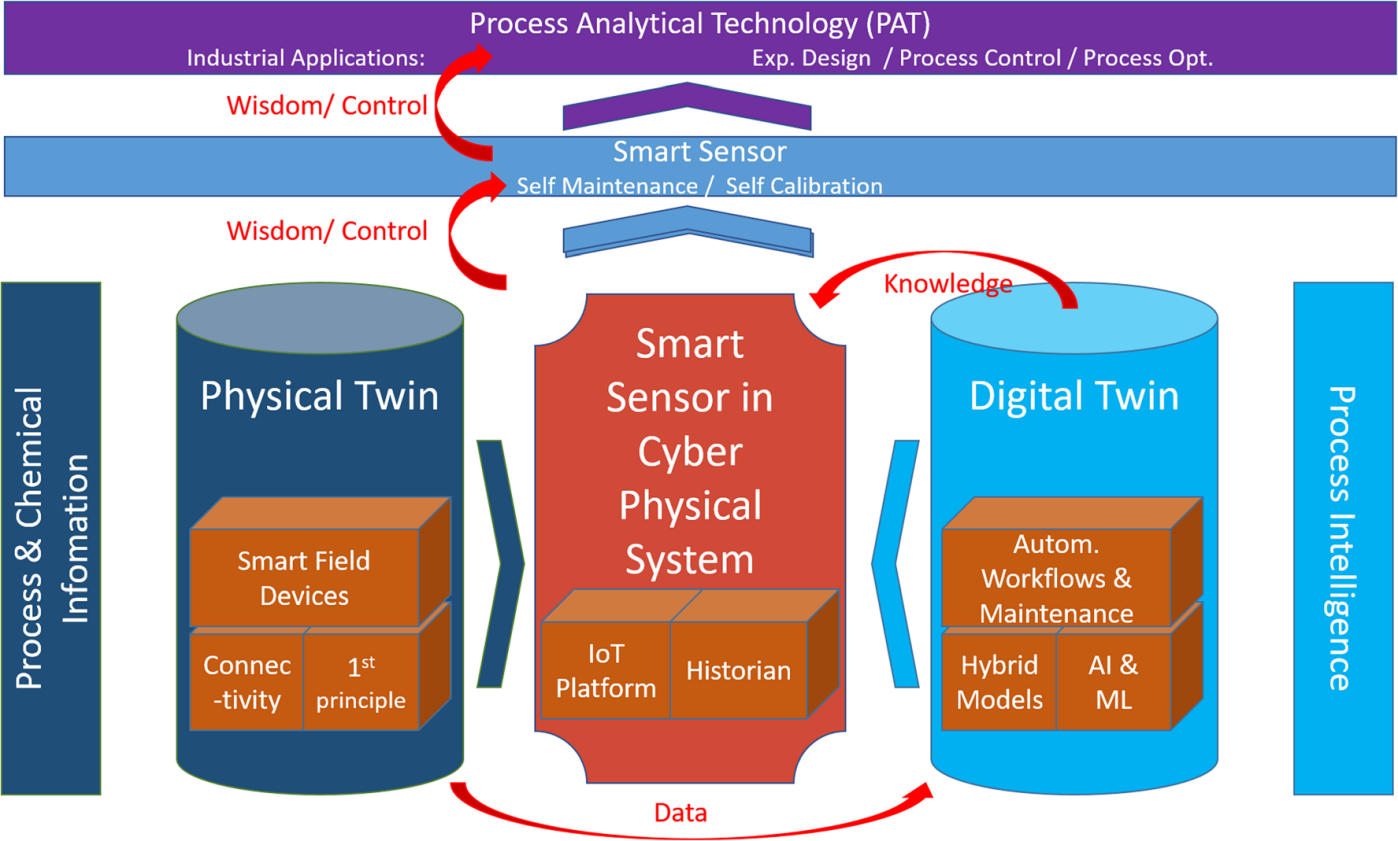
Conceptual representation of future smart sensors, consisting of a combination of elements of (1) process and chemical information (dark blue) and process intelligence (light blue). The chemical information elements feed the physical twin, the process installation, while process intelligence is implemented in the digital twin. In each of them, individual technological elements (orange) are implemented, as they will be discussed in this contribution. The smart sensor is a result of the combination of abbe elements implemented in the cyber-physical system (CPS, red) and can finally be deployed in a multitude of industrial applications in a PAT environment (top)

We are convinced that the benefits of establishing and operating smart (process) analytical labs, as mentioned in Part 1 [3] will become easier accessible via use of PAT along the whole asset life cycle together with connectivity to smart sensors. But PAT does not provide isolated measurements alone. PAT needs to be interpreted in its full definition, hence a system to design, analyze, and control the process for ensuring product quality and safety of the process [4]. Therefore, we have to broaden our perception, beyond of that we only need a sensor in the field. The sensor needs to be smart. It may be a hardware field device, a pure software element, or a combination of these—called soft sensor. Hence, the smart PAT sensor needs a combination of a chemical analytical device (elucidating the chemical and process information) and process intelligence (Fig. 1).

Thus, as our hypothesis we define the optimum smart sensor as follows:

The smart sensor measures multiple components, is self-calibrating and self-optimizing. It is easy to be integrated in the process environment – with regard to process connections and communication connectivity – and maintains its operation autonomously. In addition, it possesses process intelligence and can generate information from multi-sensor and multi-dimensional data. Integrated in a PAT environment it allows for a flexible but also target-oriented predictive control strategy and can compensate variations of the process and raw material attributes.

In this contribution, we will shortly summarize requirements for smart sensors, revisiting the needs and requirements of smart field devices and linking the gathered physical and chemical information to process intelligence and control solutions inside of digital twin environments.

## Data from the physical twin: smart field devices for chemical and process information

### Need for smart field devices

At present, innovative concepts in the field of process engineering and in particular process intensification are being promoted for the analysis and design of innovative equipment and production methods. This leads to a considerable improvement in sustainability and efficiency. Environmental performance will be improved, for example, through alternative energy conversion. Such concepts also improve hydrodynamics and heat transfer within processes.

Current research focuses on intensifying continuous processes. Compared with conventional batch processes, it allows for more intensive continuous production, the adoption of new compounds that are difficult to produce (see example in the “Knowledge from the digital twin: hybrid models, artificial intelligence and machine learning for process intelligence” section) improves product uniformity and significantly reduces the consumption of raw materials and energy. In addition, continuous processes often meet higher safety requirements, as they only produce hazardous chemicals when needed and do not have to be stored in large quantities. This is a clear advantage for plants located near or in urban areas, which is typical for most major chemical parks in Europe. Flexible (modular) chemical plants can produce multiple commodities with the same equipment, with short inter campaign downtime and fast time to market for new products. Typically, such plants are smaller in scale than basic chemical plants in batch production, but are still able to produce between kilograms and tons of specialty products daily.

A very high degree of automation is therefore a prerequisite for the realization of such benefit from intensified continuous plants. In continuous flow processes, continuous, automated measurements and strict product quality control are indispensable. If these are not readily available, there is a high potential danger of producing large volume of out-of-spec (OOS) products.

In pharmaceutical production, the agreed long-term vision is “continuous manufacturing” (CM), based on “real time release testing” (RTRT), i.e., risk-based and fully integrated QC in every process unit. For flexible production, modular facilities based on standardized architectures to be developed in the near future will enable plug-and-produce approaches suitable for small batches. This provides a flexible connection of smaller production plants, production transfer to fully autonomous plants, less user intervention, less non-productive time, and a continuous process knowledge over the product life cycle, future expertise, and a faster market entrance. It is also supposed that the costs for quality control within a CM concept will be substantially reduced at the same time.

The increasing demand for automation in general and PAT sensors in particular will changes the way PAT engineers design, install, and operate sensors in the field, especially in the context of modular plants and the reusability of respective PAT modules. A smart field device, which is capable to fulfill this, does know what its colleague devices in the smart labs—as defined in Part 1, our preceding contribution [2]—did during the process development and connects to the sensors and actuators around it. Furthermore, smart field devices have process connections, built-in safety, and regulatory specifications already implemented to ease the installation and enhance the reusability of PAT. This will increase the yield of chemical information and will significantly support the respective control strategy, if combined with process information.

### “Chemistry controls chemistry”

A production plant in the process industry usually changes the chemical and physical properties of the used substrate. Thereby, an increase in value is achieved by using knowledge how to transform raw materials into value products and goods. In chemical plants, for example, a substrate like propene is oxidized to acrolein or acrylic acid. Therefore, the chemical plant breaks and makes chemical bonds to achieve an added value. Still, control strategies within the process industry mainly focus on classical instrumentation measuring variables like pressure, temperature, level, or flow and not specific information (i.e., “chemical” such as physico-chemical properties and chemical reactions) that present the changes in chemical structure and composition. For example, measuring the functional groups that are changed by the plant (C=C, C=O, and COOH) during propene oxidation would obtain the concentration of main or by-products.

Future processes will need to be more flexible in using a broad range of raw materials, such as bio-based materials for circular economy solutions. So far, the substances added to a process show very small ranges of fluctuation in their specifications. In contrast, renewable and recycled input materials are subject to severe changes and fluctuations of the bandwidth of their properties—depending on their specific origin. However, since these raw materials are increasingly used, new requirements for PAT tools need to be met—e.g., due to fouling of measurement probes that need to be overcome [5].

The flexibility with respect to the use of raw materials is, for example, shown today in energy industry using secondary fuels or biofuels. This results in new requirements for the chemical analytics of the raw materials in discontinuous processes (e.g., incoming goods inspection) and continuous processes (e.g., material flows in pipelines and on conveyor belts).

For the chemical industries, the monitoring of chemical information is the key to “chemical” process control. A chemical factory that performs chemical reactions is controlled in a closed loop by specific information. As an example, a case study was made of a given aromatic coupling reaction step (lithiation reaction). The project challenge was to integrate a commercially available low-field NMR spectrometer from a desktop application to the complete requirements of an industrial automation environment, including accurate interpretation of measurement data [6].

Today, in process industry, spectroscopic methods are increasingly applied to measure specific chemical information online. For example, NIR spectroscopy is often applied for online analytics in the liquid phase and is an excellent industry-proven tool for a wide range of applications. The need of future flexibility demands more chemical sensors revealing comprehensive chemical information, like Raman spectroscopy, mass spectrometry, or the above-mentioned NMR spectroscopy. It will be a joint task of the PAT users, research, and equipment and software manufactures bringing these “too complex” analyzers to the field through encapsulation of the complexity by modular approaches.

### Current and future requirements to smart process sensors and actuators

Sensors are the sensing organs of industrial automation. There are presently profound developments in information and communication technology that provide great opportunities for optimized process control and added value with dedicated cross-linked communicative sensors. These types of “intelligent” sensors can provide services within a particular network and use information from there. Consequently, smart process sensors enable new innovative business models for users, device manufacturers, and service providers.

**Fig. 2:**
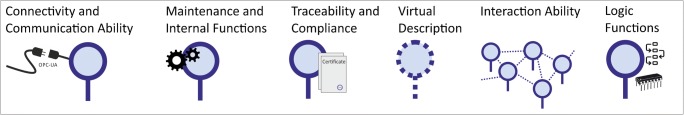
Important features of a smart analytical device, process sensor, or actuator

Recently the technology roadmap “Process Sensors 4.0” was published. It describes the necessary requirements as well as the communication capabilities of such process sensors—from simple temperature sensors to state-of-the-art technologies still being developed. Important smart features are shown in Fig. 2. Many of these requests have meanwhile been successfully and reliably realized in first case studies being evidence of excellent cooperation between users in the process industry and their device and software manufacturers in committees and joint projects.

The cost of connectivity is dropping dramatically, providing the great chance to connect people, assets, and information across the industrial enterprise. While only providing add-on information for the initial stage, future comprehensive cloud services may not require a high disposability or real-time capabilities. Though, worldwide known tech companies already demonstrated the available cloud computing power with respect to real-time data analysis—but for (process) industry, real-time transmission of complex and a large number of signals/data is a major challenge. If these are given in future, even process control tasks will be possible using cloud services, e.g., when complex computing algorithms are needed, which require more computing power than can be possibly provided by edge computing devices.

### Future requirements to communication/connectivity

In automation technology, there is currently a large number of process control systems (PCS), such as automation and control systems, operating and monitoring systems, and manufacturing execution systems for controlling a process, while process information management systems (PIMS) are used for data acquisition and evaluation. The transitions are smooth. Meaningful data acquisition takes place in PIMS together with stored process steps (“recipe”), which are called up as required and specify all raw materials, materials, and production equipment used (plant, reactors, plant equipment). In this way, deviations can be stored in the system accordingly. The database contains further fields for all relevant data from production, quality control of substances and materials, and the product.

Access to the system is ideally secured by, e.g., controlling user roles or traffic monitoring and can be logged in an audit trail if required. In critical cases, it is even today already possible (but not yet implemented as industry-standard) to only allow raw materials to enter production if their identity and specification are unique—for example by using fast fingerprint methods such as Raman spectroscopy. Of course, data safety needs to be solved as well, but is out of scope of the present contribution. In areas subject to regulatory supervision, opinions on the need to store in-process data or to document OOS results are often less sharp than those for release data [7]. From a technical point of view, a complete intermediate storage of all measured data is not always necessary if, for example, the frequency of data collection is very high. Depending on the dynamics of the process step, representative data should be stored.

How can complex process analytical devices be integrated? In the field of industrial communication, an unmanageable variety of bus systems are used to transmit complex information: Industrial Ethernet, Profinet, Modbus, AS-Interface, IO-Link, Industrial Wireless Communication—just to name a few. For some procedures, which originate from the laboratory environment, no professional bus systems are (yet) available and one is dependent on the connection of a (local) evaluation computer. This is unacceptable from the control technology point of view if no status control of the evaluation computer is installed in order to achieve the necessary robustness and to establish a safe operating point in the event of system failure.

In order to achieve this, steps were taken towards simplification and standardization, because today automation components in a plant are not at all standardized. Thus, a uniform protocol and a uniform fieldbus are required for trouble-free communication between all automation components. Meanwhile, the standard OPC Unified Architecture (OPC-UA) [8] is considered to be set and can be regarded as a small triumph of industry 4.0. Non-ethernet field buses are still dominant today against the background of a grown landscape in existing plants and the often very special requirements for power supply and explosion protection.

In the figurative sense, OPC-UA is comparable to the PDF standard or HTML standard, which define the properties of graphical objects, e.g., print products. It is also independent of manufacturers or system suppliers, programming language, operating system, or communication standard (e.g., fieldbus) and standardizes the underlying data format, e.g., for online measured values. The German Federal Office for Information Security (BSI) confirmed in 2016 that OPC-UA can be used to implement IT-safe industrial 4.0 communication [9].

Because the maintenance and operational functions are of great benefit, some innovative companies in the process industry are currently covering their plants with additional network access, mostly wireless technologies. Companies have also started to completely digitize their asset and plant plans. The question whether the high amount of information needs to be integrated in the classical pyramid automation structure or there might be another interface between the classical PCS domain and the monitoring and optimization domain is covered by the NAMUR open architecture concept (NOA) [10, 11].

## Knowledge from the digital twin: hybrid models, artificial intelligence, and machine learning for process intelligence

### Data contextualization

Beyond of the subjects on data connectivity and data integrity, as exemplified in the previous section, data need to be contextualized for holistic data science–based process analysis. Data sources contain time value pairs, and also discrete data from LIMS (Laboratory Information Management Systems) or ELN (Electronic Laboratory Notebooks), and will increasingly include 2D and 3D data from LC-MS (liquid chromatography–mass spectrometry) or image-based analytics. Tools and algorithms will need to be in place for automated feature extraction (e.g., towards chemical information) from above-mentioned data sources and unfolding of data dimensionality [12]. This will allow jointly analyzing links between density features from images to growth kinetics as an example. Feature-based analysis will also help differentiating valuable data from noise, being independent of scale and initial conditions of an individual data set. Remember: a time axis without context information is a lost axis in process data analysis.

### Between the poles of mechanistic understanding and AI

Once data are contextualized and preprocessed (see the “Smart sensor applications: the only constant is change” section) process analysis can start. Multivariate statistics such as PCA (principle component analysis); PLS (partial least squares); and LDA (latent discriminant analysis) as well as more advanced methods such as sparse and robust PLS [13] will remain the initial basis for hypothesis generation “how” features of process parameters and raw material attributes act on key performance indicators and quality attributes. However, this only solves the “how” but not the “why.” Therefore, multivariate statistics will remain as an essential hypothesis generator for advanced mechanistic exploration.

But will mechanistic understanding still be required in the age of AI (artificial intelligence)? As for the process industry, we strongly encourage to use AI first as an image of our intelligence, hence transferring our intellectual knowledge into an artificial system so that we are freeing up our minds for new challenges that require more creativity. However, AI is more: It is “any device that perceives its environment and takes actions that maximize its chance of successfully achieving its goals” [14].

Those solutions are supposed to adapt or learn autonomously to new data sets and derive new causal links between process parameters and process variables, for which the underlying algorithms ML (machine learning) and DL (deep learning) algorithms will come in to play. ML uses supervised learning algorithms [15]. This is the part in our domain knowledge, which is of highest importance when interpreting data sets with lots of parameters (*p*) with a low number of observations (*n*) (classical *n*<<*p* problem) and relates strongly to what we already do in multivariate sparse data science [16]. DL is unsupervised but demands larger data sets. Pure DL solutions in the world of PAT are attractive for smart decisions, but need clear traceability in a regulated environments and model validation gets explicitly important, as currently discussed [17].

Hence, we strongly suggest that mechanistic understanding shall be the basis before blindly dumping data into a black box of ML or DL algorithms. We would lose one more generation of process understanding. Hybrid models, as we recently defined it [18], will be the most suitable platform to capture knowledge and provide the knowledge as a hybrid model, a highly potent element of a smart sensor. We can implement this model in a cyber-physical system in order to create a digital twin.

### The huge application potential of digital twins

We have a hybrid model, what can we do with it? What do we need for its application as smart sensor? There are several dimensions of deploying a model along the product life cycle:

- Model-based experimental design for acceleration of (bio-)process development
- In combination with hard sensors/field devices for redundant measurements for enhanced process robustness
- As integration in cyber-physical systems (CPS)
- CPS-integrated digital twins allowing suggestion schemes, i.e., for control solutions, predicting events, or process optimization
- Digital twins as essential element for training via VR (virtual reality)

## The integration of the twins in cyber-physical systems

Intelligent field devices, digital communication, Internet Protocol (IP)–enabled connectivity and easy-to-use applications form the basis for the future project “Industry 4.0” and industrial internet of things (IIoT). Historically and continuously recorded data are analyzed by advanced data analysis software. This is a prerequisite for the implementation of Cyber-Physical Systems (CPS) as part of these upcoming automation concepts for the process industry. “Cyber-Physical Systems are integrations of computational models with physical processes, the real time environment. Embedded models, computers and networks monitor and control the physical processes, usually with feedback loops where physical processes affect computations and vice versa” [19].

Hence, the CPS fuses the physical and digital twin and allows its connectivity to edge computing, historians etc. This will allow real-time execution of hybrid sensors for advance feedback process control and efficient experimental design as digital twin.

Hybrid model derived process states from data gathered by smart field devices using data-driven or mechanistic links could be realized in future. Thus, these provide redundant estimates to the field devices. In future models, such kind of models will be combined with the filed devices as hybrid sensors. Hence, hybrid sensors can vice versa be used to calibrate field devices or other models in certain process states. Data science solutions on multivariate consistency check and fault diagnosis [20] of estimated and redundant measurements will need to be in place in real time.

## Smart sensor applications: the only constant is change

A very important facet of smart is when knowledge is used for applications, hence, when knowledge meets control strategies and wisdom/intelligence. Whatever will be set up in an industrial application, the change will occur soon. The change is due to change in raw material, scale, site, equipment, personnel, and many more causes.

We have to adapt field devices in its calibration, using hybrid sensor approaches, as mentioned above. Auto-calibration could be accessible via connection from the sensor-processing unit to a database that is itself connected to PIMS and LIMS. When the chemistry is known (e.g., the functional groups of a reagent and its effect on sensor signals are known, i.e., a physical understanding is given) and reference data is available—even complex sensors could perform a self-calibration.

For the transition from process analytical technology to process analysis, we will analyze data for consistency in automated data preprocessing workflows, e.g., using multivariate data-driven tools for outlier detection, diagnosis algorithms for checking calibration/model validity and drifts. We will use fingerprint tools for pattern recognition, for example robust and sparse PLS models [21].

We will maintain or even better adapt digital twins along good modelling practice using cloud-based automated workflows [22]. Self-adapting digital twins will identify process phases and process states and allow multiple models to run in real time in parallel. Data science solutions for model switching will choose the best model being part of the digital twin for the current, control, optimization, or estimation task. This can be supplemented with sematic sensors, in which expert knowledge is embedded—speeding up an implementation project of model-based real-time optimization (RTO) in the process industry [23].

However, we have to have control on the hybrid sensors we deploy, because we need to demonstrate data integrity of data sources, processing solutions as well as adaptive digital twins for the full production control strategy [24]. Therefore, to guarantee authenticity of such hybrid sensors, blockchain solutions may be suitable means for demonstrating data integrity in the future [25]. Hence, the overarching subject of model maintenance will be an own research field in the future, which will include traceability of self-adapting AI and ML solutions, important for products in the regulated process industry.

## Smart sensor facilitated PAT applications

Finally, the smart sensor represents the essential enabler for industrial applications, in which PAT plays a central role. Here we will only mention some of those applications along the product life cycle, from smart process development to robust manufacturing controls:

### Model-based experimental designs

Hybrid models are a means to measure efficiently, because fewer measurements are required. For example, quality attributes or physiological process parameters, which are difficult to measure, can be become available. With this, production can move from purely explorative designs such as design of experiments (DoE) to model-based designs. The advantages are multiple [26]: controlling based on non-measurable process parameters, reducing the amount of experiments, more efficient determination of sweet spots, avoiding non-executable process conditions due to flexible experimental boundaries.

### Digital twin based process control

CPS-integrated digital twins will enable to control complex processes on an objective function for process optimization. While in place in chemical industry since a long time, we will see multiple input multiple output control (MIMO) solutions also for processes with complex product quality attributes. The novelty will be the capability to operate the control strategy inside of the multidimensional design space and therefore benefit from the interactivity of the process parameters [27]. Apart from specific process control objectives of a certain unit operation, this will also revolutionize the overall production control strategy along the life cycle [28], enabling smooth scale-up and technology transfer.

## Conclusions

The key function of the smart sensor is to simplify their use, their maintenance as well as their calibration, supervised by digital twin intelligence and facilitated by plug-and-play integration in a CPS. This requires standardization as well as a module type re-design of process connection and connectivity. Hence, smart sensors enable concepts like self-diagnostics, self-calibration, and self-configuration/parameterization. The ingredients of a smart sensor are summarized in Table 1.

**Table 1:** Main ingredients of smart sensors

Smart sensor segment	Elements
Chemical and process information	Connectivity Chemistry controls chemistry Multiple components at once
Process intelligence	Data contextualization and holistic data analysis Workflows to generate information and understanding Knowledge capture in digital twins/AI/ML/DL
CPS, the integration element	Combined HW and SW Solutions Historian connectivity Edge computing
Smart sensor applications	IIoT platform Adaptive solutions for sensor optimization Self-maintenance/management Self-calibration
Smart sensor–facilitated PAT applications	Digital twin–based experimental design Digital twin–driven optimum control Continued process verification Golden batch controls

There is an increasing need to measure quality attributes and raw material attributes, which is enabled by smart sensors such as digital twin deployment. This also represents a basis for comprehensive use of chemistry-controlled chemistry plants.

Compared with Industry 3.0, IIoT is nothing else than integration of individual tools. The individual sensors may already exist and used. The individual PAT sensors have no IIoT 4.0 relevance without its integration/combination to a smart sensor.

To turn this into reality, we propose several levels of integration that turn individual PAT sensors into a high potent smart sensor tool. The integration needs to be flexible, because we talk of a flexible manufacturing platform for multiple products and a product life cycle approach:

- Software agility: We propose DevOps techniques, i.e., software development practices that combine software development (Dev) and information technology operations (Ops), and a DevOps mindset, as an agile approach, without gigantic deployment, test, or validation overheads. Although DevOps is successfully applied currently, especially for software as a service deployment, it is not yet fully established for IT/OT environments in all process industry segments [29].
- Data agility: SaaS (software as a service) Cloud solutions will be the future basis for data and knowledge exchange. We have to solve cyber biosecurity concerns, on technical but mainly on political level. Beyond of this, we are convinced that SaaS tools will provide the required agility of exchanging data, but also for model and digital twin life cycling for enabling smart sensors.
- Holistic data management and data analysis: We need to push data connectivity, including standardization of data interfaces and consistent data models. However, data availability is not enough. We need also to extend this to ability to holistically analyze the different data sources, integrating time value pairs, spectra, images, ELN, LIMS, and MES data. We need standardized analysis platforms and agreements on dashboards for knowledge display.
- Model and digital twin agility: We need a flexible but still validatable environment for capturing and deploying knowledge, because the models and digital twins will need to be adapted along the life cycle and still be continuously deployable in a regulated and running manufacturing environment. This includes, AI and ML, but solution should be found in all kind of hybrid solutions. Therefore, we need validated workflows for automated model development and digital twin deployment, including integrated model maintenance, model management and fault detection algorithms.
- Interdisciplinary curricula: Numerous data scientists will be required to run the factory of the future. Workforce development standardization would be useful to ensure that expectations for training and proficiency are uniform across the industry. Agreement upon a standard curricula and assessment measures would facilitate this standardization. Initiatives should be launched at universities but shall include data science schools on industrial level as well. This includes usage of virtual reality (VR) for training.
- The full potential of PAT, in combination within smart labs (as elaborated in part 1 of this series), will increase quality, reduce costs, be a clear contribution to the internet of things and accelerate time to market.

## References

[1] Meyer K, Kern S, Zientek N, Guthausen G, Maiwald M. Process control with compact NMR. TrAC Trends Anal Chem. 2016;83.

[2] Maiwald M, Gräßer P, Wander L, Zientek N, Guhl S, Meyer K, Kern S (2017). Strangers in the night—smart process sensors in our current automation landscape. Proceedings.

[3] Eisen K, Eifert T, Herwig C, Maiwald M, Current and future requirements to industrial analytical infrastructure – part 1: process analytical laboratories, Anal. Bioanal. Chem. (accepted). 10.1007/s00216-020-02420-2 PMC7072061 32060581

[4] ICH. Q8, Pharmaceutical development (R2). www.ich.org. 2009.

[5] Eifert T, Liauw MA (2016). Process analytical technology (PAT) applied to biomass valorisation: a kinetic study on the multiphase dehydration of xylose to furfural. React Chem Eng.

[6] Kern S, Wander, L, Meyer K, Guhl S, Mukkula, A.R.G., Holtkamp M., Salge M., Fleischer C., Weber N., King R., Engell S., Paul A., Remelhe M.P., Maiwald M. Flexible automation with compact NMR spectroscopy for continuous production of pharmaceuticals. Anal Bioanal Chem 2019;411:3037–3046. doi:10.1007/s00216-019-01752-y. 10.1007/s00216-019-01752-y PMC6526149 30903225

[7] U.S. Department of Health and Human Services Food and Drug Administration Center for Drug Evaluation and Research (CDER), Investigating out-of-specification test results for pharmaceutical production guidance for industry . https://wwwfdagov/regulatory-information/search-fda-guidance-documents/investigating-out-specification-test-results-pharmaceutical-production (accessed 31072019). 2006.

[8] OPC-Foundation http://www.opcfoundation.org (Accessed 28072019).

[9] OPC-UA. Security analysis by German Office for Information Security (BSI). https://opcfoundationorg/wp-content/uploads/2017/04/OPC_UA_security_analysis-OPC-F-Responses-2017_04_21pdf (Accessed 28072019).

[10] Klettner C, Tauchnitz T, Epple U, Nothdurft L, Diedrich C, Schröder T, Urbas L (2017). Namur open architecture. Atp Magazin.

[11] De Caigny J, Tauchnitz T, Becker R, Diedrich C, Schröder T, Großmann D, Urbas L (2019). NOA–Von Demonstratoren zu Pilotanwendungen. Atp Magazin.

[12] Mercier SM, Diepenbroek B, Dalm MCF, Wijffels RH, Streefland M (2013). Multivariate data analysis as a PAT tool for early bioprocess development data. J Biotechnol.

[13] Todorov V, Filzmoser P. Comparing Classical and Robust Sparse PCA. In: Kruse R., Berthold M., Moewes C., Gil M., Grzegorzewski P., Hryniewicz O. (eds) Synergies of Soft Computing and Statistics for Intelligent Data Analysis. Advances in Intelligent Systems and Computing, vol 190. Springer, Berlin, Heidelberg.

[14] Poole D*;* Mackworth A, Goebel R *(1998).* Computational intelligence: a logical approach*. New York: Oxford University Press.* ISBN 978-0-19-510270-3*.*

[15] The Industrial Internet of Things Volume T3, https://www.iiconsortium.org/pdf/IIC_Industrial_Analytics_Framework_Oct_2017.pdf accessed: 2019-12-24

[16] Osborne MR, Presnell B, Turlach BA (2000). A new approach to variable selection in least squares problems. IMA J Numer Anal.

[17] Proposed Regulatory Framework for Modifications to Artificial Intelligence/Machine Learning (AI/ML)-Based Software as a Medical Device (SaMD) - Discussion Paper and Request for Feedback, https://www.regulations.gov/document?D=FDA-2019-N-1185-0001, assessed 2019-12-24

[18] Solle D, Hitzmann B, Herwig C, Remelhe PM, Ulonska S, Wuerth L, Prata A, Steckenreiter T (2017). Between the poles of data-driven and mechanistic modeling for process operation. Chem Ing Tech.

[19] Lee EA.. Cyber physical systems: design challenges. EECS Dep., University of California, Berkeley. http://www2.eecs.berkeley.edu/Pubs/TechRpts/2008/EECS-2008-8pdf (accessed 19012018). 2008.

[20] Jiang B, Zhub X, Huanga D, Paulsonb JA, Braatz RD (2016). A combined canonical variate analysis and Fisher discriminantanalysis (CVA–FDA) approach for fault diagnosis. Comput Chem Eng.

[21] Todorov V, Filzmoser P. Comparing classical and robust sparse PCA. Synergies of Soft Computing and Statistics for Intelligent Data Analysis 2013.

[22] Kroll P, Hofer A, Stelzer IV, Herwig C (2017). Workflow to set up substantial target-oriented mechanistic process models in bioprocess engineering. Process Biochem.

[23] Müller D, Dercks B, Nabati E, Blazek M, Eifert T, Schallenberg J, Dadhe K (2017). Real-time optimization in the chemical processing industry. Chem Ing Tech.

[24] Herwig C, Wölbeling C, Zimmer T (2017). A holistic approach to production control. Pharm Eng.

[25] Steinwandter V, Herwig C. Provable data integrity in the pharmaceutical industry based on version control systems and the blockchain. PDA Journal. 2019. 10.5731/pdajpst.2018.009407. 10.5731/pdajpst.2018.009407 30770485

[26] Kroll P, Hofer A, Ulonska S, Kager J, Herwig C (2017). Model-based methods in the biopharmaceutical process lifecycle. Pharm Res.

[27] Zahel T, Hauer S, Mueller E, Murphy P, Abad S, Vasilieva E (2017). Integrated process modeling—a process validation life cycle companion. Bioeng..

[28] ICH. Technical and regulatory considerations for pharmaceutical product lifecycle management. Q12. 2017.

[29] Steinwandter VB, Daniel Herwig C. Data science tools and applications on the way to Pharma 4.0. Drug Discov Today. 2019. 10.1016/j.drudis.2019.06.005. 10.1016/j.drudis.2019.06.005 31207205

